# The effect of alcohol consumption on all-cause mortality in 70-year-olds in the context of other lifestyle risk factors: results from the Gothenburg H70 birth cohort study

**DOI:** 10.1186/s12877-023-04227-z

**Published:** 2023-08-28

**Authors:** Felicia Ahlner, Hanna Falk Erhag, Lena Johansson, Jessica Samuelsson, Hanna Wetterberg, Madeleine Mellqvist Fässberg, Margda Waern, Ingmar Skoog

**Affiliations:** 1 Neuropsychiatric Epidemiology Unit, Institute of Neuroscience and Physiology, Department of Psychiatry and Neurochemistry, Sahlgrenska Academy at the University of Gothenburg, Wallinsgatan 6, 431 41 Gothenburg, SE Sweden; 2 Centre for Ageing and Health (AgeCap) at the University of Gothenburg, Gothenburg, Sweden; 3 Region Västra Götaland, Sahlgrenska University Hospital, Psychiatry, Addiction Clinic, Gothenburg, Sweden; 4 Region Västra Götaland, Sahlgrenska University Hospital, Psychosis Clinic, Gothenburg, Sweden; 5 Region Västra Götaland, Sahlgrenska University Hospital, Psychiatry, Cognition and Old Age Psychiatry Clinic, Gothenburg, Sweden

**Keywords:** Alcohol, 70-year-olds, Septuagenarians, Risk factors, Unhealthy behaviors, Mortality, Premature death, Epidemiology, H70

## Abstract

**Background:**

In this study, we examined the effect of alcohol, as well as the combined effect of seven lifestyle factors, on all-cause mortality in older adults (baseline age 70 years).

**Methods:**

Data was derived from the population-based Gothenburg H70 Birth Cohort study, including 1124 participants from the 2014–16 examination. Risk consumption was defined as  > 98 g alcohol per week, and hazardous drinking was based on the Alcohol Use Disorders Identification Test-Consumption questionnaire (AUDIT-C). Cox regression models were used to examine the individual effect of alcohol consumption, as well as the combined effect of seven lifestyle risk factors (high alcohol consumption, lifetime smoking, unhealthy Body Mass Index, insufficient physical activity, sedentary behavior, insufficient/prolonged sleep, unhealthy dietary pattern) on all-cause mortality.

**Results:**

During a mean follow-up of 7.7 years, 81 (7.2%) participants died. Neither risk consumption nor hazardous drinking were associated with elevated mortality, but hazardous drinking was associated with an increased risk of mortality in those with insufficient physical activity. Those with at least five lifestyle risk factors had an increased all-cause mortality compared to those fulfilling criteria for a maximum of one lifestyle risk factor. High alcohol consumption showed a relatively minor impact on this risk,﻿ while physical activity and unhealthy dietary pattern had an independent effect on mortality.

**Conclusions:**

In this particular sample, there was no independent effect of alcohol on the risk of 8-year all-cause mortality. However, an interaction effect of physical activity was observed. It may be that high alcohol consumption per se is less important for mortality among older adults. However, a combination of several unhealthy lifestyle behaviors was linked to a substantial increase in the risk of mortality in Swedish older adults. Also, it has to be emphasized that high alcohol consumption may have other adverse health effects apart from mortality among older adults.

**Supplementary Information:**

The online version contains supplementary material available at 10.1186/s12877-023-04227-z.

## Background

Alcohol use significantly impacts population health, contributing to unintentional injuries, diseases, and mortality [1]. Among older adults, consumption rates have increased in recent years [2–4]. We have previously reported a rising prevalence of at-risk consumption (≥ 100 g of pure alcohol per week) among 70-year-olds, with rates increasing from 16 to 45% in men and from 0.5 to 24% in women between 1976 and 2016 [2]. Among 85-year-olds, the prevalence rose from 10 to 25% in men and from 2 to 9% in women between 1986 and 2015 [5]. Globally, the increasing trend of alcohol consumption has resulted in a 25.9% rise in alcohol-related mortality between 2007 and 2017 among adults aged 70 years and older [6]. Given the process of population aging, it is reasonable to expect that the consequences of alcohol consumption will become a growing public health issue.

Age-related physiological changes, such as decreased total body water and increased body fat, contribute to higher blood alcohol concentration and prolonged alcohol effects in older adults compared to younger individuals [3, 7]. Studies have linked heavy alcohol consumption (variously defined) to all-cause mortality in middle-aged or older adults [8–10]. However, there is inconsistent evidence regarding the effects of lower alcohol consumption levels. The observed J-shaped associations between alcohol consumption and specific conditions, such as cetrain cardiovascular diseases and diabetes, in some epidemiological studies have faced criticizism due to biases like abstainer bias and insufficient control for confounding factors [11, 12]. Growing evidence suggests that the risk of all-cause mortality increases with higher alcohol consumption [13]. However, it is suggested that the risk of adverse effects associated with lower levers of alcohol consumption is relatively lower for older populations compared to younger individuals [14].

Apart from alcohol use, numerous other lifestyle factors significantly influence overall health, and a healthy lifestyle has been associated with a reduced risk of mortality in both general and middle-aged (≥ 50 years) populations [15–18]. Few studies have examined the impact of alcohol consumption on mortality risk in older adults, considering other lifestyle risk factors. In addition, limited studies have investigated the effect of sedentary behavior and sleep, which are emerging risk factors for mortality. We aimed to examine the association between alcohol consumption and all-cause mortality during 8-years of follow-up among 70-year-olds living in Gothenburg, Sweden. In addition, we examined the combined effect of alcohol and six other lifestyle risk factors on overall mortality risk among current drinkers, utilizing a 7-item lifestyle risk score.

## Methods

### Sample

The data for this study was obtained from the baseline examination of the 1944 birth cohort in the Gothenburg H70 Birth Cohort Study (H70 studies), which are multidisciplinary epidemiological studies focused on examining the health of older adults. In order to yield representative samples, participants are systematically selected from the Swedish population register based on specific birth dates. Between 2014 and 2016, a total of 1203 participants underwent examination, resulting in a response rate of 72.2%. The sampling procedure and characteristics of the study sample have been previously described in detail [19]. All participants underwent a comprehensive general health examination, which included face-to-face interviews, physical examinations, self-reported questionnaires, and a battery of tests. For the purpose of this study, 64 participants were excluded due to missing alcohol data, and an additional 15 participants were excluded due to dementia [20], leaving an analytic sample of 1124 participants.

Compared to those excluded (*n* = 79), participants in the analytic sample (*n* = 1124) had a higher level of education (*p* < 0.001). There was no statistically significant difference in the median age at death between the analytic sample and those excluded (*p* = 0.463).

### Measures

#### Alcohol consumption

Alcohol intake was assessed at baseline using two different assessments: total weekly consumption (grams of pure alcohol per week) and the 3-item Alcohol Use Disorders Identification Test-Consumption (AUDIT-C) [21]. Information on weekly alcohol consumption was obtained through face-to-face interviews conducted by psychiatric research nurses. Participants reported the weekly frequency of alcohol intake and average weekly consumption of specific beverage types (i.e., beer, wine, and spirits) during the past month. Total weekly consumption in grams of pure alcohol was calculated based on the amount of each beverage and the reference alcohol content. Risk consumption was defined as  > 98 g per week (g/week), following the guidelines from the National Institute on Alcohol Abuse and Alcoholism (NIAAA) for individuals aged 65 years and older [22]. In further analyses, > 98 g/week was divided into two groups:  > 98–195 g/week and  ≥ 196 g/week.

The AUDIT-C was utilized to identify hazardous drinking among current drinkers. The AUDIT-C is a widely used screening tool for self-reported past-year alcohol consumption and consists three questions: *“How often do you have a drink containing alcohol?”*, *“How many standard drinks containing alcohol do you have on a typical day?”*, and *“How often do you have six or more drinks on one occasion?”* Each question offers five predetermined response options valued from 0 to 4 points, resulting in a total score ranging from 0 to 12 points. In this study, cutoffs of  ≥ 5 for men and  ≥ 4 for women were employed, based on a recent validation of the AUDIT-C for hazardous drinking in community-dwelling adults aged 60 and over [23].

Alcohol abstainers were identified during the face-to-face interview and were classified as lifetime abstainers (never drinkers) or former drinkers (participants who had quit drinking).

#### Covariates

Information on covariates was obtained through self-ratings, questionnaires, and interviews conducted by trained clinical staff during the baseline examination in 2014–16. Country of birth (born in Sweden or born outside Sweden), education (≤ primary or  ≥ secondary), smoking (lifetime or never) and religiousness (yes or no) were obtained by self-report. The burden of somatic disease was assessed using a comorbidity score that included four chronic non-communicable diseases or categories of diseases (i.e., cardiovascular disease (CVD), liver disease, cancer, and diabetes) with major contributions to global total or alcohol-attributable deaths [1, 24]. CVD diagnoses encompassed atrial fibrillation, myocardial infarction, heart failure, angina pectoris, hypertension, intermittent claudication, stroke, and transient ischemic attack (TIA). These diagnoses were based on self- or proxy-reported symptoms, supplemented with additional information from electrocardiography (ECG) and register data for some conditions. Hypertension was defined as having a blood pressure of  ≥ 140 mmHg systolic or  ≥ 90 mmHg diastolic, or taking antihypertensive medication. The details of the diagnostic procedures have been described previously [25]. History of liver disease, cancer, and diabetes were self-reported, excluding cases of youth-onset liver disease (< 18 years).

Depression (classified as minor according to DSM-III-R and major according to DSM-IV) was determined based on information regarding depressive symptoms obtained from semi-structured psychiatric examinations, as described elsewhere [26]. Due to the highly functionally independent sample, functional dependence was defined as a Barthel Index score of less than 100, indicating limited abilities in activities of daily living (ADL) [27]. Poor self-rated health was identified by “Poor” or “Fair” responses on the five-point scale question in the 36-Item Short-Form Health Survey (SF-36) [28].

### Lifestyle risk score

The combined effect of alcohol consumption and six other lifestyle risk factors on mortality risk was evaluated using a 7-item lifestyle risk score. This score included alcohol consumption, smoking status, Body Mass Index (BMI), physical activity, sedentary time, sleep, and dietary pattern. Definitions of alcohol consumption and smoking are provided above.

BMI  < 23 or  ≥ 31 was classified as unhealthy based on guidelines for adults aged 65 and over [29]. Physical activity was assessed using the International Physical Activity Questionnaire-Short Form (IPAQ-SF) [30]. Insufficient physical activity was defined as not meeting global and national physical activity guidelines for individuals aged 65 and over (≥ 150 min/week of moderate activities,  ≥ 75 min/week of vigorous activities, or a combination moderate-vigorous activity at a similar level) [31, 32]. Sedentary time exceeding 7 h per day was classified as unfavorable based on cutoffs suggested in previous meta-analyses [33, 34]. Insufficient/prolonged sleep (< 7 or  ≥ 9 h/night) was defined based on general recommendations for adults aged 65 and over [35]. To assess dietary pattern, a score measuring adherence to the Mediterranean diet was constructed based on information collected during a diet history interview. The methods for constructing the score are described in detail elsewhere [19, 36–38]. Consumption exceeding the sex-specific median for healthy food groups or falling below the median for less healthy food groups generated 1 point. The sum score ranged from 0 to 8, with scores of 0–4 indicating low adherence to the Mediterranean diet, representing a less healthy dietary pattern.

For each of the seven lifestyle risk factors, unhealthy patterns generated 1 point, while healthy patterns generated 0 points. Thus, the total lifestyle risk score ranged from 0 to 7, with higher scores reflecting a greater number of lifestyle risk factors.

### Mortality

Date of death was obtained from the Swedish Tax Agency, which included all deaths in Sweden. Follow-up continued until death or, at the latest, February 9, 2022. Participants who emigrated (*n* = 4) during the follow-up period were censored at the date of departure. For one participant with an unknown date of death, July 2 (midway through the calendar year) was assigned.

### Statistical analysis

Sample characteristics are presented as numbers, median, range, and percentages, and differences between categorical data were analyzed using Pearson’s chi-square test and Fisher’s exact test.

Cox regression models using age as a time scale were used to analyze the relationship between alcohol consumption categories and all-cause mortality in three different models. Schoenfeld residuals indicated that the proportional hazard assumption was met. Results are presented as hazard ratios (HRs) and 95% confidence intervals (CIs) in three models. Model 1 included sex as a covariate. Model 2 included sex, country of birth, education, smoking, and religion. Model 3 further included somatic burden score, depression, functional independence, and self-rated health as potential mediating factors. Potential confounding factors were selected a priori based on previous literature [15, 39–41]. Due to the small sample size and few events among lifetime abstainers and former drinkers, abstainers were not included in the Cox regression analyses.

We examined the interaction between alcohol consumption and two potential moderating factors (sex and education) in relation to the risk of mortality using Model 1. Additionally, we examined the potential interaction effects between alcohol consumption and each of the six other lifestyle factors (one at a time) to evaluate potential effect modification.

The combined effect of alcohol and six other lifestyle risk factors on mortality was investigated using a 7-tem risk score. The association between lifestyle risk score and all-cause mortality was analyzed using Model 1–3, excluding smoking. Thus, Model 1 included sex, in Model 2 country of birth, education, and religion were added. Model 3 further included somatic burden score, depression, functional independence, and self-rated health. Due to the small number of participants (< 6%) with a lifestyle risk score of 0, 5, 6, or 7, we combined lifestyle risk scores 0 and 1, and lifestyle risk scores 5, 6, and 7, respectively. In order to increase the sample size with a lifestyle risk score, missing data on one lifestyle factor was considered acceptable, except for alcohol consumption, which was the primary focus of the present study.

We examined the interaction of alcohol and each of the remaining six lifestyle factors in the lifestyle risk score sample. We also checked for potential effect modification by sex and education in the association between alcohol consumption and mortality in the lifestyle risk score sample. Model 1 was used for all interaction effect tests.

Sensitivity analyses were conducted by excluding one lifestyle risk factor at a time to examine the individual effect of each factor and determine whether the association was driven by any of the included factors. Models 1–3 were performed by adding the excluded factor as covariate.

Primary analyses used definitions of risk consumption in accordance with NIAAA guidelines (> 98 g/week). In secondary analyses, all analyses were repeated using hazardous drinking based on the AUDIT-C score (≥ 5 for men,  ≥ 4 for women). Analyses were conducted using IBM SPSS version 28 or Stata version 15.

## Results

Among the 1124 participants at baseline, the majority were current drinkers (93.4%), followed by former drinkers (3.7%) and lifetime abstainers (2.8%). Table 1 shows baseline sample characteristics and differences between deceased and surviving participants. During an average follow-up of 7.73 years (standard deviation 1.10), 81 participants died (7.2%). At baseline, 339 participants (dead/alive: 24/315) consumed > 98 g per week, and 290 participants (dead/alive: 23/267) met the criteria for hazardous drinking, with an overlap in 204 cases (dead/alive: 16/188). A higher proportion of men were risk consumers compared to women (*p* = 0.013, Table 1). Among the 79 current drinkers who died during the study period, the median weekly consumption was 46 g at baseline, compared to 63 g among those who were still alive at the end of follow-up.

**Table 1:** Characteristics of 70-year-olds followed for 8 years, by sex and vital status (end of study)

	Total sample (*n* = 1124)					
	Men (517/1124)	Women (607/1124)	*p*-value^a^	Alive (1043/1124)	Dead (81/1124)	*p*-value^a^
	% (no. cases/total)	% (no. cases/total)		% (no. cases/total)	% (no. cases/total)	
Drinking status			.234			.221^b^
Lifetime abstainer	1.9 (10/517)	3.6 (22/607)		3.1 (32/1043)	0.0 (0/81)	
Former drinker	3.9 (20/517)	3.6 (22/607)		3.8 (40/1043)	2.5 (2/81)	
Current drinker	94.2 (487/517)	92.8 (563/607)		93.1 (971/1043)	97.5 (79/81)	
Weekly consumption among current drinkers						.706
0–98 g	53.6 (261/487)	20.1 (113/563)	< .001	67.5 (656/971)	69.6 (55/79)	
> 98 g	46.4 (226/487)	79.9 (450/563)		32.4 (315/971)	30.4 (24/79)	
AUDIT-C score^c^ among current drinkers			.189			.757
Non-hazardous drinking	70.4 (343/487)	74.1 (417/563)		72.5 (704/971)	70.9 (56/79)	
Hazardous drinking	29.6 (144/487)	25.9 (146/563)		27.5 (267/971)	29.1 (23/79)	
Born outside Sweden	15.9 (82/517)	12.9 (78/605)	.156	13.4 (140/1041)	24.7 (20/81)	.005
≥ Secondary education	83.9 (434/517)	88.0 (534/607)	.052	86.4 (901/1043)	82.7 (67/81)	.357
Lifetime smoker	62.8 (324/516)	60.6 (368/607)	.457	61.3 (639/1042)	65.4 (53/81)	.464
Being religious	20.5 (105/511)	27.8 (165/594)	.005	24.3 (249/1024)	25.9 (21/270)	.746
Somatic disease						
Continuous, median (range)	1.0 (3)	1.0 (3)	.065	1.0 (3)	1.0 (3)	.979
Depression (minor or major)	6.4 (33/515)	10.8 (65/604)	.010	8.7 (90/1039)	10.0 (8/80)	.683
ADL < 100	12.6 (65/514)	15.4 (92/596)	.183	13.4 (138/1030)	23.8 (19/80)	.010
Poor self-rated health	13.8 (71/515)	17.5 (105/600)	.090	14.6 (151/1034)	30.9 (25/81)	< .001

^a^Pearson Chi-Square test

^b^Fisher’s Exact Test

^c^Cut-off score for hazardous drinking: ≥ 5 for men,  ≥ 4 for women

Compared to those alive at the end of the study, those who died were more often born outside Sweden (*p* = 0.005), were more often functionally dependent (*p* = 0.010), and had poorer self-rated health (*p* < 0.001). The majority (*n* = 79) of deaths occurred among those who were current drinkers at baseline.

No significant associations were found regarding the risk of mortality between individuals who consumed  ≤ 98 g/week [reference] and those who consumed  > 98 g/week, as well as between non-hazardous drinkers [reference] and hazardous drinkers (Table 2).

**Table 2:** Baseline alcohol consumption and all-cause mortality in a H70 study sample 2014–2022 (*n* = 1124; baseline age 70)

Alcohol consumption categories	Model 1		Model 2		Model 3	
	HR (95% CI)	*p*-value	HR (95% CI)	*p*-value	HR (CI)	*p*-value
Gram per week						
0–98 g /week	1		1		1	
> 98 g /week	0.81 (0.49–1.33)	.403	0.91 (0.55–1.52)	.722	0.98 (0.58–1.64)	.923
> 98 to < 196 g /week	0.63 (0.33–1.19)	.155	0.73 (0.38–1.39)	.332	0.76 (0.40–1.47)	.418
≥ 196 g /week	1.17 (0.61–2.23)	.642	1.28 (0.66–2.49)	.470	1.37 (0.69–2.69)	.368
AUDIT-C score ^a^						
Non-hazardous drinking	1		1		1	
Hazardous drinking	1.08 (0.67–1.76)	.746	1.20 (0.73–1.98)	.470	1.31 (0.79–2.18)	.300

Cox regression models using age as time scale, presented as Hazard ratios (HR) and 95% Confidence intervals (CI)

Model 1: Adjusted for sex

Model 2: Adjusted for sex, country of birth, education, smoking, and religion

Model 3: Adjusted for sex, country of birth, education, smoking, religion, somatic disease, depression, self-rated health, and functional dependence

^a^Cut-off score for hazardous drinking:  ≥ 5 for men,  ≥ 4 for women

An interaction was observed between hazardous drinking and physical activity in relation to mortality (*p* = 0.005, Model 1). Among those with insufficient physical activity, hazardous drinkers had an increased risk of mortality compared to non-hazardous drinkers (Model 2 HR: 3.13, 95% CI 1.25–7.81, Table 3). This association did not reach statistical significance after additional adjusting for somatic disease, depression, self-rated health, and functional dependence (Model 3 HR: 2.59, 95% CI 0.98–6.82, Table 3). However, estimates followed expected attenuation for Model 3, and *p*-value was close to significance level (*p* = 0.054). Among those with sufficient physical activity, there was no significant association between alcohol consumption and mortality (Model 3 HR: 0.69, 95% CI 0.31–1.52, Table 3). There were no other interactions observed in the alcohol-mortality association.

**Table 3:** AUDIT-C score^a^ and all-cause mortality in current drinkers (*n* = 1050; baseline age 70), by physical activity

	**Model 1**		**Model 2**		**Model 3**	
	HR (95% CI)	*p*-value	HR (95% CI)	*p*-value	HR (95% CI)	*p*-value
*Sufficient physical activity*^*b*^						
Non-hazardous drinking	1		1		1	
Hazardous drinking	0.56 (0.26–1.19)	.132	0.63 (0.29–1.38)	.247	0.69 (0.31–1.52)	.359
*Insufficient physical activity*^*c*^						
Non-hazardous drinking	1		1		1	
Hazardous drinking	**2.83 (1.15–6.97)**	**.023**	**3.13 (1.25–7.81)**	**.015**	2.59 (0.98–6.82)	.054

Cox regression models using age as time scale, presented as Hazard ratios (HR) and 95% Confidence intervals (CI)

Model 1: Adjusted for sex

Model 2: Adjusted for sex, country of birth, education, and religion

Model 3: Adjusted for sex, country of birth, education, religion, somatic disease, depression, self-rated health, and functional dependence

^a^Cut-off score for hazardous drinking:  ≥ 5 for men,  ≥ 4 for women

^b^Sufficient physical activity =  ≥ 150 min/week of moderate activities or  ≥ 75 min of vigorous activity

^c^Insufficient physical activity =  < 150 min/week of moderate activities or  < 75 min of vigorous activity

### Alcohol consumption in the context of a lifestyle risk score

Table 4 shows baseline characteristics for participants with a lifestyle risk score (*n* = 898). Compared to those with no lifestyle risk score (*n* = 226), those with a lifestyle risk score had higher education (*p* < 0.001). There was no statistically significant difference regarding median age at death between the groups (*p* = 0.951).

**Table 4:** Baseline characteristics in participants with a lifestyle risk score^a^ (*n* = 898; baseline age 70)

	0–1 (*n* = 207)	2 (*n* = 291)	3 ( *n* = 241)	4 (*n* = 106)	5–7 (*n* = 53)	*p*-value^*b*^
	% (no. cases/total)	% (no. cases/total)	% (no. cases/total)	% (no. cases/total)	% (no. cases/total)	
Men	41.5 (86/207)	45.4 (132/291)	49.8 (120/241)	52.8 (56/106)	58.5 (31/53)	.101
Born in Sweden	84.5 (175/207)	85.9 (250/291)	90.0 (217/241)	87.7 (217/241)	87.7 (93/106)	.303
≥ Secondary education						
Being religious	29.8 (61/205)	25.0 (72/288)	19.9 (48/241)	15.1 (16/106)	15.1 (8/53)	.012
*Factors in lifestyle risk score*						
Alcohol consumption						
0 = 0–98 g/week	93.7 (194/207)	75.3 (219/291)	57.3 (138/241)	34.9 (37/106)	28.3 (15/53)	< .001
1 = > 98 g/week	6.3 (13/207)	24.7 (72/291)	42.7 (103/241)	65.1 (69/106)	71.7 (38/53)	
Smoking status						< .001
0 = Never smoker	73.9 (153/207)	42.6 (124/291)	19.5 (47/241)	13.2 (14/106)	0.0 (0/53)	
1 = Lifetime smoker	26.1 (54/207)	57.4 (167/291)	80.5 (194/241)	86.8 (92/106)	100.0 (53/53)	
Body Mass Index						< .001
0 = Normal weight	84.9 (174/205)	66.9 (194/290)	59.6 (143/240)	37.7 (40/106)	30.8 (16/52)	
1 = Unhealthy weight	15.1 (31/205)	33.1 (96/290)	40.4 (97/240)	62.3 (66/106)	69.2 (36/52)	
Physical activity						< .001
0 = Sufficient	99.0 (205/207)	91.7 (266/290)	81.3 (196/241)	67.9 (72/106)	32.1 (17/53)	
1 = Insufficient	1.0 (2/207)	8.3 (24/290)	18.7 (45/241)	32.1 (34/106)	67.9 (36/53)	
Sedentary time						< .001
0 = ≤ 7 h/d	98.5 (193/196)	92.2 (248/269)	87.5 (182/208)	74.7 (68/91)	51.1 (23/45)	
1 = > 7 h/d	1.5 (3/196)	7.8 (21/269)	12.5 (26/208)	25.3 (23/91)	48.9 (22/45)	
Sleep duration						
0 = 7-8 h/d	88.4 (183/207)	77.0 (224/291)	54.6 (131/240)	41.5 (44/106)	15.1 (8/53)	< .001
1 = < 7 or ≥ 9 h/d	11.6 (24/207)	23.0 (67/291)	45.4 (109/240)	58.5 (62/106)	84.9 (45/53)	
Dietary pattern						< .001
0 = Adherence to Mediterranean diet	79.7 (122/153)	42.3 (99/234)	25.5 (51/200)	17.9 (17/95)	11.5 (6/52)	
1 = Low adherence to Mediterranean diet	20.3 (31/153)	57.7 (135/234)	74.5 (149/200)	82.1 (78/95)	88.5 (46/52)	

^a^Lifestyle risk score based on alcohol consumption (0–98 g/week, > 98 g/week), smoking status (never, lifetime), Body Mass Index (23–30, < 23 or ≥ 31), physical activity (≥ 150 min/week of moderate activities or ≥ 75 min of vigorous activity, < 150 min/week of moderate activities or < 75 min of vigorous activity), sedentary time (≤ 7 h/day, > 7 h/day), sleep (7–8 h/night, < 7 or ≥ 9 h/night) and dietary pattern (dietary pattern score ≥ 5, dietary pattern score ≤ 4)

^b^Pearson Chi-Square test

A total of 207 had no or one lifestyle risk factor (dead/alive: 12/195), 638 had two to four lifestyle risk factors (dead/alive: 41/597), and 53 had at least five lifestyle risk factors (dead/alive: 9/44). The most prevalent lifestyle risk factor was lifetime smoking (62.4%), followed by unhealthy dietary pattern (59.8%), unhealthy weight (36.5%), insufficient/prolonged sleep (34.2%), risk consumption of alcohol (32.9%), insufficient physical activity (15.7%), and sedentary behavior (> 7 h/day) (11.7%). At the end of follow-up, 62 (6.9%) out of 898 participants with a lifestyle risk score had died.

In the lifestyle risk score sample (*n* = 898), lifestyle risk score 5–7 was associated with increased risk of all-cause mortality in all models, with highest estimates after adjustments for sex, country of birth, education, and religion (Model 2 HR: 3.76, 95% CI 1.51–9.40, Table 5). After additional adjustments for somatic disease, depression, self-rated health, and functional independence, the hazard ratio decreased (Model 3 HR: 3.11, 95% CI 1.11–8.67, Table 5).

**Table 5:** Lifestyle risk score and all-cause mortality (*n* = 898; baseline age 70)

	**Model 1**		**Model 2**		**Model 3**	
	HR (95% CI)	*p*-value	HR (95% CI)	*p*-value	HR (95% CI)	*p*-value
**Lifestyle risk score with alcohol consumption based on weekly consumption**^**a**^						
Lifestyle risk score 0–1	1		1		1	
Lifestyle risk score 2	0.77 (0.35–1.69)	.511	0.79 (0.36–1.73)	.554	0.87 (0.39–1.96)	.741
Lifestyle risk score 3	1.39 (0.67–2.87)	.376	1.55 (0.75–3.23)	.240	1.67 (0.78–3.61)	.189
Lifestyle risk score 4	1.34 (0.54–3.28)	.528	1.44 (0.57–3.61)	.438	1.40 (0.54–3.64)	.496
Lifestyle risk score 5–7	**3.18 (1.33–7.64)**	**.010**	**3.76 (1.51–9.40)**	**.005**	**3.11 (1.11–8.67)**	**.030**
**Lifestyle risk score with alcohol consumption based on AUDIT-C score**^**b**^						
Lifestyle risk score 0–1	1		1		1	
Lifestyle risk score 2	0.85 (0.39–1.84)	.684	0.90 (0.42–1.95)	.789	1.00 (0.44–2.18)	1.00
Lifestyle risk score 3	1.44 (0.71–2.91)	.314	1.56 (0.77–3.18)	.221	1.62 (0.77–3.41)	.208
Lifestyle risk score 4	0.97 (0.37–2.56)	.951	1.21 (0.45–3.25)	.708	1.28 (0.46–3.56)	.641
Lifestyle risk score 5–7	**4.19 (1.83–9.56)**	** < .001**	**5.51 (2.29–13.29)**	** < .001**	**3.96 (1.43–10.95)**	**.008**

Cox regression models using age as time scale, presented as Hazard ratios (HR) and 95% Confidence intervals (CI)

Model 1: Adjusted for sex

Model 2: Adjusted for sex, country of birth, education, and religion

Model 3: Adjusted for sex, country of birth, education, religion, somatic disease, depression, self-rated health, and functional dependence

^a^Lifestyle risk score based on alcohol consumption (0–98 g/week, > 98 g/week), smoking status (never, lifetime), Body Mass Index (23–30, < 23 or ≥ 31), physical activity (≥ 150 min/week of moderate activities or ≥ 75 min of vigorous activity, < 150 min/week of moderate activities or < 75 min of vigorous activity), sedentary time (≤ 7 h/day, > 7 h/day), sleep (7–8 h/night, < 7 or ≥ 9 h/night) and dietary pattern (dietary pattern score ≥ 5, dietary pattern score ≤ 4)

^b^Lifestyle risk score based on alcohol consumption (AUDIT-C score, cut-off score for hazardous drinking: ≥ 5 for men, ≥ 4 for women), smoking status (never, lifetime), Body Mass Index (23–30, < 23 or ≥ 31), physical activity (≥ 150 min/week of moderate activities or ≥ 75 min of vigorous activity, < 150 min/week of moderate activities or < 75 min of vigorous activity), sedentary time (≤ 7 h/day, > 7 h/day), sleep (7–8 h/night, < 7 or ≥ 9 h/night) and dietary pattern (dietary pattern score ≥ 5, dietary pattern score ≤ 4)

In the total lifestyle risk score sample, there was an interaction between risk consumption and educational level in relation to mortality (*p* = 0.007, Model 1), i.e., there was a tendency that risk consumption was associated with increased risk of mortality in those with only primary education or less. There were no other interactions with risk consumption observed in the lifestyle risk score sample.

The associations between lifestyle risk score 5–7 and risk of mortality remained in secondary analyses using AUDIT-C as a measure of alcohol consumption (Table 5). Compared to definitions of risk consumption in primary analyses, estimates were stronger in all models using hazardous drinking (Model 3 HR: 3.96, 95% CI 1.43–10.95, Table 5). In the total lifestyle risk score sample, an interaction was observed between hazardous drinking and physical activity in relation to mortality (*p* = 0.003, Model 1), i.e., there was a tendency that hazardous drinking was associated with higher mortality in those with insufficient physical activity. There were no other interactions with hazardous drinking observed in the lifestyle risk score sample.

### Sensitivity analyses

Analyses were repeated with exclusion of one lifestyle risk factor at a time. The positive association with mortality remained in the fully adjusted model after exclusion of alcohol consumption, smoking, and dietary pattern from the lifestyle risk score (Model 3, Table 6). The magnitude of the association between the lifestyle risk score and mortality was stronger when excluding alcohol consumption, attenuated when excluding smoking or dietary pattern, and no longer statistically significant when excluding BMI, physical activity, sedentary time, or sleep duration (Table 6).

**Table 6:** Lifestyle risk score (alcohol consumption based on weekly consumption)^a^ with one excluded factor and all-cause mortality (*n* = 898; baseline age 70)

	**Model 1**		**Model 2**		**Model 3**	
	HR (95% CI)	*p*-value	HR (95% CI)	*p*-value	HR (95% CI)	*p*-value
*Excluding alcohol use*						
Lifestyle risk score 0–1	1		1		1	
Lifestyle risk score 2	1.28 (0.63–2.60)	.491	1.36 (0.67–2.76)	.399	1.56 (0.75–3.25)	.234
Lifestyle risk score 3	1.65 (0.78–3.47)	.189	1.68 (0.80–3.54)	.171	1.60 (0.73–3.52)	.243
Lifestyle risk score 4–6	**3.63 (1.69–7.76)**	** < .001**	**4.14 (1.89–9.11)**	** < .001**	**3.57 (1.52–8.36)**	**.003**
*Excluding smoking*						
Lifestyle risk score 0–1	1		1		1	
Lifestyle risk score 2	1.69 (0.91–3.15)	.097	1.78 (0.95–3.32)	.070	1.77 (0.93–3.38)	.083
Lifestyle risk score 3	1.65 (0.75–3.62)	.211	1.77 (0.80–3.94)	.160	1.63 (0.72–3.71)	.245
Lifestyle risk score 4–6	**3.60 (1.61–8.03)**	**.002**	**3.96 (1.74–8.99)**	**.001**	**3.01 (1.26–7.24)**	**.014**
*Excluding BMI*						
Lifestyle risk score 0–1	1		1		1	
Lifestyle risk score 2	0.97 (0.48–1.95)	.933	0.95 (0.47–1.92)	.887	0.97 (0.46–2.02)	.932
Lifestyle risk score 3	1.64 (0.84–3.21)	.145	1.90 (0.96–3.77)	.067	**2.10 (1.04–4.25)**	**.040**
Lifestyle risk score 4–6	1.97 (0.89–4.37)	.096	**2.38 (1.03–5.50)**	**.043**	2.13 (0.86–5.25)	.101
*Excluding physical activity*						
Lifestyle risk score 0–1	1		1		1	
Lifestyle risk score 2	0.78 (0.38–1.62)	.501	0.87 (0.42–1.82)	.714	0.93 (0.44–1.99)	.859
Lifestyle risk score 3	1.30 (0.65–2.63)	.459	1.42 (0.70–2.89)	.332	1.62 (0.77–3.40)	.205
Lifestyle risk score 4–6	1.56 (0.72–3.35)	.259	1.75 (0.79–3.86)	.165	1.40 (0.59–3.35)	.447
*Excluding sedentary time*						
Lifestyle risk score 0–1	1		1		1	
Lifestyle risk score 2	0.85 (0.39–1.87)	.692	0.87 (0.39–1.91)	.721	0.97 (0.43–2.20)	.948
Lifestyle risk score 3	1.44 (0.68–3.04)	.344	1.62 (0.76–3.45)	.213	1.67 (0.75–3.71)	.208
Lifestyle risk score 4–6	1.82 (0.79–4.21)	.161	1.86 (0.79–4.33)	.153	1.65 (0.68–4.01)	.272
*Excluding sleep duration*						
Lifestyle risk score 0–1	1		1		1	
Lifestyle risk score 2	1.03 (0.48–2.21)	.932	1.08 (0.50–2.32)	.845	1.21 (0.55–2.67)	.631
Lifestyle risk score 3	1.08 (0.47–2.44)	.862	1.27 (0.55–2.96)	.574	1.26 (0.52–3.04)	.605
Lifestyle risk score 4–6	2.27 (0.87–5.97)	.095	2.47 (0.92–6.63)	.073	2.22 (0.79–6.27)	.132
*Excluding dietary pattern*						
Lifestyle risk score 0–1	1		1		1	
Lifestyle risk score 2	0.82 (0.37–1.85)	.634	0.86 (0.38–1.95)	.718	0.89 (0.39–2.04)	.788
Lifestyle risk score 3	1.44 (0.62–3.33)	.395	1.43 (0.61–3.34)	.413	1.39 (0.59–3.30)	.452
Lifestyle risk score 4–6	**2.92 (1.28–6.64)**	**.011**	**3.19 (1.36–7.49)**	**.008**	**2.72 (1.12–6.57)**	**.027**

Cox regression models using age as time scale, presented as Hazard ratios (HR) and 95% Confidence intervals (CI)

Model 1: Adjusted for the excluded factor and sex

Model 2: Adjusted for the excluded factor, sex, country of birth, education, smoking, and religion

Model 3: Adjusted for the excluded factor, sex, country of birth, education, smoking, religion, somatic disease, depression, self-rated health, and functional dependence

^a^Lifestyle risk score based on alcohol consumption (0–98 g/week, > 98 g/week), smoking status (never, lifetime), Body Mass Index (23–30,  < 23 or  ≥ 31), physical activity (≥ 150 min/week of moderate activities or ≥ 75 min of vigorous activity,  < 150 min/week of moderate activities or  < 75 min of vigorous activity), sedentary time (≤ 7 h/day,  > 7 h/day), sleep (7–8 h/night,  < 7 or  ≥ 9 h/night) and dietary pattern (dietary pattern score  ≥ 5, dietary pattern score  ≤ 4)

When using AUDIT-C cutoffs for alcohol consumption, the association was attenuated when excluding alcohol consumption, smoking, and dietary pattern, with less attenuation in exclusion of alcohol consumption (Table 7). There was no significant association between lifestyle risk score and risk of mortality when excluding BMI, physical activity, sedentary time, and sleep from the lifestyle risk score (Model 3, Table 7).

**Table 7:** Lifestyle risk score (alcohol consumption based on AUDIT-C score^a^) with one excluded factor and all-cause mortality (*n* = 898; baseline age 70)

	**Model 1**		**Model 2**		**Model 3**	
	HR (95% CI)	*p*-value	HR (95% CI)	*p*-value	HR (95% CI)	*p*-value
*Excluding alcohol use*						
Lifestyle risk score 0–1	1		1		1	
Lifestyle risk score 2	1.28 (0.63–2.60)	.491	1.36 (0.67–2.76)	.339	1.56 (0.75–3.25)	.234
Lifestyle risk score 3	1.65 (0.78–3.47)	.189	1.68 (0.80–3.54)	.171	1.60 (0.73–3.52)	.243
Lifestyle risk score 4–6	**3.63 (1.69–7.76)**	** < .001**	**4.14 (1.89–9.11)**	** < .001**	**3.57 (1.52–8.36)**	**.003**
*Excluding smoking*						
Lifestyle risk score 0–1	1		1		1	
Lifestyle risk score 2	1.38 (0.75–2.56)	.300	1.42 (0.77–2.63)	.262	1.41 (0.75–2.66)	.291
Lifestyle risk score 3	1.25 (0.57–2.77)	.576	1.48 (0.66–3.34)	.340	1.30 (0.57–2.96)	.526
Lifestyle risk score 4–6	**4.27 (2.00–9.12)**	** < .001**	**5.35 (2.38–11.98)**	** < .001**	**3.83 (1.59–9.22)**	**.003**
*Excluding BMI*						
Lifestyle risk score 0–1	1		1		1	
Lifestyle risk score 2	1.11 (0.57–2.15)	.760	1.11 (0.57–2.16)	.757	1.09 (0.55–2.19)	.799
Lifestyle risk score 3	1.27 (0.63–2.57)	.500	1.60 (0.77–3.30)	.202	1.82 (0.87–3.84)	.114
Lifestyle risk score 4–6	**2.38 (1.14–4.97)**	**.021**	**3.05 (1.40–6.64)**	**.005**	**2.79 (1.22–6.39)**	**.015**
*Excluding physical activity*						
Lifestyle risk score 0–1	1		1		1	
Lifestyle risk score 2	1.05 (0.51–2.13)	.901	1..18 (0.58–2.40)	.658	1.25 (0.60–2.62)	.553
Lifestyle risk score 3	1.41 (0.70–2.87)	.340	1.46 (0.71–2.97)	.303	1.50 (0.71–3.18)	.289
Lifestyle risk score 4–6	1.63 (0.75–3.54)	.217	2.00 (0.89–4.48)	.093	1.44 (0.60–3.50)	.417
*Excluding sedentary time*						
Lifestyle risk score 0–1	1		1		1	
Lifestyle risk score 2	1.25 (0.59–2.63)	.565	1.25 (0.59–2.65)	.554	1.46 (0.67–3.16)	.343
Lifestyle risk score 3	1.37 (0.63–2.97)	.422	1.48 (0.68–3.24)	.319	1.49 (0.65–3.41)	.342
Lifestyle risk score 4–6	1.89 (0.82–4.40)	.138	2.01 (0.86–4.73)	.109	1.94 (0.79–4.78)	.151
*Excluding sleep duration*						
Lifestyle risk score 0–1	1		1		1	
Lifestyle risk score 2	1.12 (0.58–2.17)	.746	1.18 (0.61–2.30)	.620	1.24 (0.62–2.48)	.545
Lifestyle risk score 3	1.25 (0.62–2.54)	.532	1.50 (0.73–3.09)	.272	1.62 (0.77–3.42)	.202
Lifestyle risk score 4–6	**2.22 (1.03–4.79)**	**.040**	**2.85 (1.26–6.41)**	**.012**	2.37 (0.96–5.89)	.063
*Excluding dietary pattern*						
Lifestyle risk score 0–1	1		1		1	
Lifestyle risk score 2	0.90 (0.41–1.98)	.790	0.90 (0.40–2.01)	.797	0.89 (0.39–2.00)	.773
Lifestyle risk score 3	1.18 (0.47–2.94)	.723	1.18 (0.47–2.97)	.729	1.12 (0.44–2.87)	.815
Lifestyle risk score 4–6	3.48 (1.57–7.71)	.002	3.86 (1.68–8.88)	.001	2.98 (1.23–7.20)	.015

Model 1: Adjusted for the excluded factor and sex

Model 2: Adjusted for the excluded factor, sex, country of birth, education, and religion

Model 3: Adjusted for the excluded factor, sex, country of birth, education, religion, somatic disease, depression, self-rated health, and functional dependence

^a^Lifestyle risk score based on alcohol consumption (AUDIT-C score, cut-off score for hazardous drinking:  ≥ 5 for men,  ≥ 4 for women), smoking status (never, lifetime), Body Mass Index (23–30,  < 23 or  ≥ 31), physical activity (≥ 150 min/week of moderate activities or  ≥ 75 min of vigorous activity,  < 150 min/week of moderate activities or  < 75 min of vigorous activity), sedentary time (≤ 7 h/day,  > 7 h/day), sleep (7–8 h/night,  < 7 or  ≥ 9 h/night) and dietary pattern (dietary pattern score  ≥ 5, dietary pattern score  ≤ 4). Cox regression models using age as time scale, presented as Hazard ratios (HR) and 95% Confidence intervals (CI)

In the lifestyle risk score sample, insufficient physical activity and unhealthy dietary pattern were independently associated with increased mortality risk (Model 3, Table 8). There was an independent effect of insufficient or prolonged sleep on mortality when adjusting for only sex (Model 1, Table 8). However, associations of sleep did not remain after additional adjustments in Model 2 and Model 3.

**Table 8:** Association between each lifestyle risk factor and all-cause mortality (*n* = 898, baseline age 70)

			Model 1		Model 2		Model 3	
Lifestyle risk factor	Definition	n	HR (95% CI)	*p*-value	HR (95% CI)	*p*-value	HR (95% CI)	*p*-value
Alcohol consumption	0–98 g /week	603	1		1		1	
	> 98 g/week	295	0.85 (0.45–1.49)	.570	0.98 (0.55–1.75)	.952	1.07 (0.60–1.91)	.823
	Non-hazardous drinking ^a^	642	1		1		1	
	Hazardous drinking ^a^	256	0.96 (0.55–1.68)	.881	1.10 (0.62–1.95)	.744	1.21 (0.68–2.17)	.512
Smoking status	Never smoker	338	1		1		1	
	Lifetime smoker	560	1.16 (0.68–1.96)	.594	1.10 (0.64–1.87)	.729	1.09 (0.631.89)	.761
Body Mass Index	Normal weight	567	1		1		1	
	Unhealthy weight	326	1.49 (0.89–2.49)	.126	1.49 (0.89–2.49)	.129	1.45 (0.86–2.48)	.167
Physical activity ^b^	Sufficient	756	1		1		1	
	Insufficient	141	**2.31 (1.33–4.01)**	**.003**	**2.44 (1.39–4.28)**	**.002**	**2.24 (1.25–4.03)**	**.007**
Sedentary time	≤ 7 h/d	714	1		1		1	
	> 7 h/d	95	1.55 (0.75–3.18)	.234	1.58 (0.77–3.26)	.212	1.62 (0.77–3.39)	.205
Sleep duration	7–8 h/night	590	1		1		1	
	< 7 or ≥ 9 h/night	307	**1.68 (1.01–2.79)**	**.046**	1.61 (0.97–2.68)	.068	1.46 (0.86–2.49)	.159
Diet ^c^	Healthy dietary pattern	295	1		1		1	
	Unhealthy dietary pattern	439	**2.31 (1.14–4.68)**	**.021**	**2.32 (1.14–4.72)**	**.021**	**2.14 (1.05–4.37)**	**.037**

Model 1: Adjusted for sex

Model 2: Adjusted for sex, country of birth, education, and religion

Model 3: Adjusted for sex, country of birth, education, religion, somatic disease, depression, self-rated health, and functional dependence

^a^Defined by AUDIT-C sum score: Hazardous drinking =  ≥ 5 for men, ≥ 4 for women

^b^Physical activity: Sufficient = at least 150 min of moderate activity, at least 75 min per week of vigorous activities, or a combination moderate-vigorous activity at a similar level

^c^Diet: Healthy dietary pattern = sum score 4–8 in Mediterranean diet score (ranging from 0–8)

## Discussion

We found no association between risk consumption or hazardous drinking and 8-year all-cause mortality in this population-based study of 70-year-olds. However, there was an interaction with physical activity, i.e., hazardous drinking was associated with mortality in individuals with insufficient physical activity, while no association was observed among those with sufficient physical activity. When combining alcohol consumption and six other lifestyle risk factors, those with at least five lifestyle risk factors had an increased risk of mortality. The most important risk factors for mortality were dietary pattern and physical activity, while risk consumption or hazardous drinking had a relatively minor impact on the risk of death. Findings may be due to the complex relationship between alcohol consumption and health noted in observational studies, where moderate consumption has been associated with beneficial effects on some conditions [42, 43], and increased risk in a dose–response fashion for others [13].

A study conducted in Norway among individuals aged 65 years and over has previously reported no association between alcohol consumption and mortality risk [44]. In contrast, several studies have observed an association between alcohol consumption and mortality in general [45–47], middle-aged (aged  ≥ 40 years) [8, 9, 48–51], and older populations (aged  ≥ 60 years) [10, 52, 53]. However, a recent meta-analysis found that the population-level risk of adverse health effects of alcohol consumption was lower for older populations compared to younger [14]. Despite increasing levels of alcohol consumption among older adults in recent years [2], the absolute amounts consumed are higher in younger age groups [13], which may partly explain discrepancies among studies with different sample age. In addition, competing causes of death increases with age, making the effect of alcohol consumption less evident, which may also explain our results.

Other explanations for disparate results may include how alcohol consumption was assessed and categorized, the composition and size of study samples (e.g., health status, and disease frequency), study design (e.g., time of follow-up), study context (e.g. time period, income level of country, alcohol prices and policy, cultural context, religion), and which confounders were controlled for [13, 14, 54]. The recall period for self-reported alcohol consumption varies among studies and may contribute to disparate findings. It has been suggested that a 12-month recall is optimal for linking alcohol consumption with adverse health effects. Shorter reference periods may not adequately capture irregular drinkers, seasonal variations in alcohol consumption, or periods of heavy alcohol use [55, 56]. Additionally, the duration of the study period and timing of data collection (i.e., historical time period) can also affect alcohol consumption. Moreover, investigating different birth cohorts with different levels of alcohol consumption and other cohort-specific characteristics (e.g., health status, general mortality rate) may result in inconsistencies among studies.

In our sample of older adults, sufficient physical activity mitigated the association between alcohol consumption and the risk of mortality. To date, only a few studies have investigated whether levels of physical activity can counteract the mortality risks associated with alcohol consumption [57, 58]. Similar to our findings, a British study utilizing eight population-based surveys of individuals aged 40 years and above found that sufficient physical activity attenuated the risk of all-cause mortality among individuals who consumed alcohol below hazardous levels [58]. Furthermore, there is evidence that strongly suggests that engaging in physical activity is linked to a decreased risk of all-cause mortality [59, 60].

Our study is one of few that examines the combined effect of mulitple lifestyle risk factors on mortality risk in older adults. We identified only two studies of adults aged 65 and over that utilized a combination of lifestyle factors similar to those included in our study [61, 62]. These studies are based on data from the Chinese Longitudinal Healthy Longevity Survey, with a follow-up of at least 10 years. One of the studies reported a 1.3 fold higher risk of all-cause mortality among those exhibiting six unhealthy behaviors (alcohol consumption, smoking, BMI, physical activity, sleep, consumption of fruit/vegetables) [61]. The other study employed a 5-item score (alcohol consumption, smoking, BMI, physical activity, diet) and found a decreased mortality risk among participants who adhered to healthy behavior in all items [62].

Increased risk associated with a higher lifestyle risk score has also been observed in studies with younger samples conducted in Australia (age  ≥ 45 years) and Norway (age 20–69 years), with an average follow-up of 6.1 and 14.1 years, respectively [39, 63]. Both studies utilized lifestyle risk scores based on six comparable unfavorable lifestyle risk factors (alcohol consumption, lifetime smoking, physical inactivity, sedentary behavior, insufficient/prolonged sleep, unhealthy diet) and found a five to six times higher risk for individuals with the highest lifestyle risk score. Additionally, the Australian study reported an even higher risk among participants aged 65–79 years at baseline [39]. However, the estimates in these studies are higher than in the present study (HR 3.11), possibly due to variations in sample characteristics (e.g., sex, age, educational level), study context, study design, covariate adjustment, and factors included in the lifestyle risk score.

In the present study, the exclusion of risk consumption from the lifestyle risk score resulted in an increased effect size, while exclusion of other risk factors were associated with decrease in effect size. This indicate that risk consumption of alcohol a less detrimental risk factor. However, hazardous drinking contributed to increased risk in our study, as estimates for the combined effect of all seven risk factors was greater than estimates when hazardous drinking was excluded. A weaker association after excluding alcohol from the lifestyle score have previously been found in a Korean population (aged  ≥ 19) [40].

There are several limitations that require further discussion. First, although the study sample was systematically selected and had a high response rate, we cannot exclude the possibility of differences between the study sample and the target population. The presence of selection bias in this study might have led to the inclusion of individuals with lower alcohol consumption, better health and healthier lifestyles. As a result, this bias could potentially underestimated the true impact of alcohol consumption, as well as the combined effect of several lifestyle risk factors, on mortality risk.

Second, due to survival bias associated with high alcohol consumption, it is reasonable to believe that chronic heavy drinkers, to a larger extent than other groups, may have died before the age of 70. The lack of association between alcohol consumption and mortality may, therefore, be due to selective survival, and our findings should not be generalized to younger age groups.

Third, the findings may have been influenced by homogeneity in terms of sample age. A wider age span, including older ages, might have yielded different results.

Fourth, some subgroups were small and had a few number of deaths, which may lead to false negative findings. However, during the 8 years of follow-up, independent associations with mortality were found for other lifestyle factors (i.e., insufficient physical activity, insufficient/prolonged sleep, and dietary pattern), indicating fair statistical power to detect relevant differences.

Fifth, information on alcohol consumption and other exposures was collected at baseline only. If we had had the possibility to track changes in exposures over time, the results might have been more precise.

Sixth, alcohol consumption was self-reported, which may underestimate the true figures. However, self-reported alcohol measures have shown a good level of reliability and validity [64, 65]. Moreover, we used average weekly consumption data corresponding to past month consumption, without considering annual variations or lifetime alcohol use. This might have affected our findings. However, we also analyzed associations using AUDIT-C, an instrument with a 12-months recall period.

Finally, we used all-cause mortality instead of cause-specific mortality, due to lack of statistical power when diving mortality into subtypes. Additionally, it is important to note that the mortality rate alone may not fully capture the negative health outcomes associated with alcohol consumption, as other factors can also have significant impact on overall well-being. For example, we have previously reported higher rates of depression and liver disease among 70-year-olds with highest weekly consumption [41].

The present study has several strengths. First, the results are based on a fairly large sample that were systematically selected from a defined geographical area, and the response rate was high. Second, mortality data was derived from the Swedish Tax Agency, ensuring high-quality coverage of all deaths in Sweden. Third, influences of alcohol consumption and lifestyle risk score on mortality risk were systematically investigated using different definitions of alcohol consumptio, and conducting multiple analyses (i.e., stratified analyses, secondary analyses, and sensitivity analyses). These efforts were made to explore the possibility of type II errors (false negative findings). Fourth, we included seven lifestyle risk factors in the lifestyle risk score, including two emerging risk factors (sedentary time and sleep). The items in the lifestyle risk score were carefully selected and assessed using validated measurements administrated by trained research health professionals. Moreover, appropriate cutoffs based on previous literature were utilized to categorize the included factors. Finally, associations were adjusted for multiple carefully selected potential confounders and were sequentially included in models to evaluate the mediating effect.

## Conclusion

In this population-based study of 70-year-olds, we found no significant association between risk consumption or hazardous drinking and all-cause mortality over an eight-year period. However, among individuals with insufficient physical activity, hazardous drinking was linked to increased mortality. Furthermore, having more than five lifestyle risk factors was associated with higher all-cause mortality, with alcohol consumption being a less important factor. Our results expand on previous research by examining the combined impact of unhealthy behaviors in an older population, while also considering sleep and sedentary behavior as emerging lifestyle risk factors for mortality. These findings provide evidence for population interventions and offer guidance on healthy lifestyle recommendations for older adults. It should be noted that high alcohol consumption may lead to other health consequences beyond the risk of mortality in this age group.

### Supplementary Information

**Additional file 1: Supplementary Table 1.** Phi correlation coefficient among dichotomous health covariates in the baseline sample (*n*=1124). **Supplementary Table 2. **Phi correlation coefficient among lifestyle risk factors in the lifestyle risk score sample (*n*=898). 

## Data Availability

The data used in this article can be made available by the corresponding author upon request.
