# Supplementary material for: The effect of alcohol consumption on all-cause mortality in 70-year-olds in the context of other lifestyle risk factors: results from the Gothenburg H70 birth cohort study
Source: BMC Geriatr. 2023 Aug 28;23:523. doi: 10.1186/s12877-023-04227-z (PMC10464006; doi:10.1186/s12877-023-04227-z)
Supplement: Supplementary file 1 — Additional file 1: Supplementary Table 1. Phi correlation coefficient among dichotomous health covariates in the baseline sample (n=1124). Supplementary Table 2. Phi correlation coefficient among lifestyle risk factors in the lifestyle risk score sample (n=898). [file 12877_2023_4227_MOESM1_ESM.docx]

**ADDITIONAL FILE 1 – Supplementary Tables 1-2**

| **Supplementary Table 1.** Phi correlation coefficient among dichotomous health covariates in the baseline sample (n=1124) | | | | | | | | |
| --- | --- | --- | --- | --- | --- | --- | --- | --- |
|  |  | CVD | Liver disease | Diabetes | Cancer | Depression | Functional independence | Self-rated health |
| Total | CVD | 1 |  |  |  |  |  |  |
|  | Liver disease | -.011 | 1 |  |  |  |  |  |
|  | Diabetes | **.105***** | -.050 | 1 |  |  |  |  |
|  | Cancer | .006 | .015 | .024 | 1 |  |  |  |
|  | Depression | .050 | .004 | .012 | .034 | 1 |  |  |
|  | Functional independence | .017 | .041 | **.067*** | .026 | .052 | 1 |  |
|  | Self-rated health | .049 | -.020 | **.135***** | **.078**** | **.215***** | **.211***** | 1 |
| Men | CVD | 1 |  |  |  |  |  |  |
|  | Liver disease | .004 | 1 |  |  |  |  |  |
|  | Diabetes | .074 | .004 | 1 |  |  |  |  |
|  | Cancer | -.014 | -.018 | -.022 | 1 |  |  |  |
|  | Depression | **.097*** | .035 | .051 | .074 | 1 |  |  |
|  | Functional independence | -.005 | .051 | .041 | .011 | .019 | 1 |  |
|  | Self-rated health | .012 | .010 | .080 | .055 | **.243** | **.262***** | 1 |
| Women | CVD | 1 |  |  |  |  |  |  |
|  | Liver disease | -.022 | 1 |  |  |  |  |  |
|  | Diabetes | **.132***** | **-.098*** | 1 |  |  |  |  |
|  | Cancer | .019 | .038 | .068 | 1 |  |  |  |
|  | Depression | .026 | -.009 | -.010 | .011 | 1 |  |  |
|  | Functional independence | .036 | .035 | **.097*** | .041 | .069 | 1 |  |
|  | Self-rated health | **.018*** | -.039 | **.194***** | **.102*** | **.193***** | **.171***** | 1 |
| ^*^*p*≤0.05, ^**^*p*≤0.01, ^***^*p*≤0.001 | | | | | | | | |

| **Supplementary Table 2.** Phi correlation coefficient among lifestyle risk factors in the lifestyle risk score sample (n=898) | | | | | | | | |
| --- | --- | --- | --- | --- | --- | --- | --- | --- |
|  |  | Alcohol consumption | Smoking | Body Mass Index | Physical activity | Sedentary time | Sleep | Dietary pattern |
| Total | Alcohol consumption | 1 |  |  |  |  |  |  |
|  | Smoking | **.103**** | 1 |  |  |  |  |  |
|  | Body Mass Index | -.049 | -.050 | 1 |  |  |  |  |
|  | Physical activity | **.076*** | **.083*** | -.016 | 1 |  |  |  |
|  | Sedentary time | **.092**** | .040 | -.015 | **.113***** | 1 |  |  |
|  | Sleep | .015 | .052 | .017 | .043 | .012 | 1 |  |
|  | Dietary pattern | -.030 | .**099**** | -.021 | **.132***** | -.035 | .022 | 1 |
| Men | Alcohol consumption | 1 |  |  |  |  |  |  |
|  | Smoking | **.098*** | 1 |  |  |  |  |  |
|  | Body Mass Index | .016 | -.025 | 1 |  |  |  |  |
|  | Physical activity | .026 | **.095*** | -.031 | 1 |  |  |  |
|  | Sedentary time | .026 | .071 | -.008 | **.127*** | 1 |  |  |
|  | Sleep | .001 | .076 | .082 | **.100*** | .073. | 1 |  |
|  | Dietary pattern | .006 | .092 | .002 | **.139*** | -.098 | .055 | 1 |
| Women | Alcohol consumption | 1 |  |  |  |  |  |  |
|  | Smoking | **.099*** | 1 |  |  |  |  |  |
|  | Body Mass Index | -.017 | -.062 | 1 |  |  |  |  |
|  | Physical activity | **.106*** | .068 | .015 | 1 |  |  |  |
|  | Sedentary time | **.152**** | .004 | -.008 | **.086** | 1 |  |  |
|  | Sleep | -.002 | .026 | -.020 | -.022 | -.067 | 1 |  |
|  | Dietary pattern | -.066 | **.105*** | -.041 | **.127*** | .023 | -.005 | 1 |
| ^*^*p*≤0.05, ^**^*p*≤0.01, ^***^*p*≤0.001 | | | | | | | | |
